# Impact of lidocaine on hemodynamic and respiratory parameters during laparoscopic appendectomy in children

**DOI:** 10.1038/s41598-022-18243-3

**Published:** 2022-08-18

**Authors:** Maciej Kaszyński, Barbara Stankiewicz, Krzysztof Jakub Pałko, Marek Darowski, Izabela Pągowska-Klimek

**Affiliations:** 1 Department of Pediatric Anesthesiology and Intensive Care, Medical University of Warsaw University Clinical Centre, 63A Żwirki i Wigury St., 02-091 Warsaw, Poland; 2 Department of Modeling and Supporting of Internal Organs Functions, Nalecz Institute of Biocybernetics and Biomedical Engineering, Polish Academy of Sciences, 4 Trojdena St., 02-109 Warsaw, Poland

**Keywords:** Physiology, Drug discovery, Drug safety, Paediatric research, Medical research, Randomized controlled trials

## Abstract

We assessed the influence of systemic lidocaine administration on ventilatory and circulatory parameters, and the pneumoperitoneum impact on the cardiopulmonary system during a laparoscopic appendectomy in children. A single-center parallel single-masked randomized controlled study was carried out with 58 patients (3–17 years). Intravenous lidocaine bolus of 1.5 mg/kg over 5 min before induction of anesthesia followed by lidocaine infusion at 1.5 mg/kg/h intraoperatively. Respiratory system compliance (C, C/kg), P_peak_-PEEP and Pulse rate (Pulse), systolic, diastolic and mean blood pressure (NBP_s_, NBP_d_, NBP_m_), assessed in the Lidocaine and Control group, at the: beginning (P_1_), minimum lung compliance (P_2_) and at the end of surgery (P_3_) were compared. The respiratory/hemodynamic parameters did not differ between the groups at any stage of operation. Blood Pressure and P_peak_-PEEP were significantly higher at the P_2_ compared to P_1_ and P_3_ stages (P < 0.001, 1 − β ≥ 0.895) that correlated with lung compliance changes: C/kg vs. NBP_s_ and P_peak_-PEEP (− 0.42, − 0.84; P < 0.001); C vs. Pulse and P_peak_-PEEP (− 0.48, − 0.46; P < 0.001). Although an increase in intraabdominal pressure up to 12(15) mmHg causes significant changes in hemodynamic/respiratory parameters, there appears to be no risk of fatal reactions in 1E, 2E ASA patients. Systemic lidocaine administration doesn’t alleviate circulatory/respiratory alterations during pneumoperitoneum. No lidocaine related episode of anaphylaxis, systemic toxicity, circulatory disturbances or neurological impairment occurred.

**ClinicalTrials.gov**: 22/03/2019.

**Trial registration number**: NCT03886896.

## Introduction

According to the results from our Previous study^1^, intravenous lidocaine infusion during laparoscopic appendectomy results in a reduction of the intraoperative requirement for opioids in children. Due to opioid-sparing effect, patients are Protected from specific complications like Prolonged recovery after anesthesia, respiratory complications, Prolonged duration of ileus, postoperative nausea and vomiting, itching, urine retention. Systemic lidocaine administration appeared to be a safe modality, which is consistent with other trials on this topic^2–9^.

Laparoscopic surgery offers numerous advantages, such as: reduction of intraoperative metabolic stress response, less intraoperative bleeding, reduction of postoperative pain, better cosmetic results, enhanced recovery, reduction in hospital stay, less postoperative wound infection^10,11^. However, due to the creation of pneumoperitoneum, certain physiological alterations must be taken into account during anesthesia for this specific surgical technique.

Both the respiratory and circulatory system are affected by the increase in intraabdominal Pressure. A decrease in pulmonary compliance is observed, along with various changes in hemodynamic parameters. Episodes of hypertension, hypotension, dysrhythmias, and even cardiac arrest have been reported. This study was undertaken in recognition of the clinical significance of mentioned complications and hypothetically Protective Properties of lidocaine.

## Methods

### Study design

The study was conducted in the Pediatric Teaching Hospital at the University Clinical Centre of the Medical University of Warsaw, Poland.

In this single-blind randomized controlled trial, children were randomly assigned to two groups according to the use of intraoperative intravenous lidocaine infusions to compare the hemodynamic and respiratory reaction at the different stages of anesthesia. The study was conducted between March 2019 and January 2020. Patients were enrolled between 26/03/2019 and 15/01/2020.

In accordance with the current Polish law and the Declaration of Helsinki, the study was approved by the Ethics Committee of the Medical University of Warsaw (KB/24/2019).

The primary trial was registered at the US National Institutes of Health: NCT03886896. The date of first posted on ClinicalTrials.gov: 22/03/2019. The data presented currently were not reported in the original publication^1^, as they represented neither the primary nor the secondary outcomes of the above trial.

Nalecz Institute of Biocybernetics and Biomedical Engineering, Polish Academy of Sciences (IBIB PAS) was involved in data analysis, results Presentation and discussion.

### Study population

This paper focuses on the influence of lidocaine infusions on the circulatory and respiratory system. Since the outcomes analyzed in this report required different input data from those originally investigated^1^, only participants with a complete set of the necessary details were included. Therefore the number of participants, and their demographic and clinical characteristics are different from those Presented in the Primary trial^1^.

Children Presenting for laparoscopic appendectomy to be anesthetized by physicians involved in the study were assessed for eligibility criteria.

The inclusion criteria are listed below:

- Age between 18 months and 18 years;
- ASA physical status class 1E, 2E, 3E;
- Patients undergoing laparoscopic appendectomy.

The exclusion criteria are listed below:

- Allergy to local anesthetics or contraindications for the use of lidocaine;
- ASA physical status class 4 or higher;
- Severe cardiovascular disease;
- Preoperative bradycardia;
- Preoperative atrioventricular block;
- Renal failure;
- Chronic treatment with analgesics;
- Legal guardians’ refusal.

Researchers spoke to the parents or legal guardians and informed them about the study. They described the potential risks and benefits of the Procedure, discussed questions and concerns, and then obtained written informed consent.

**Figure 1:**
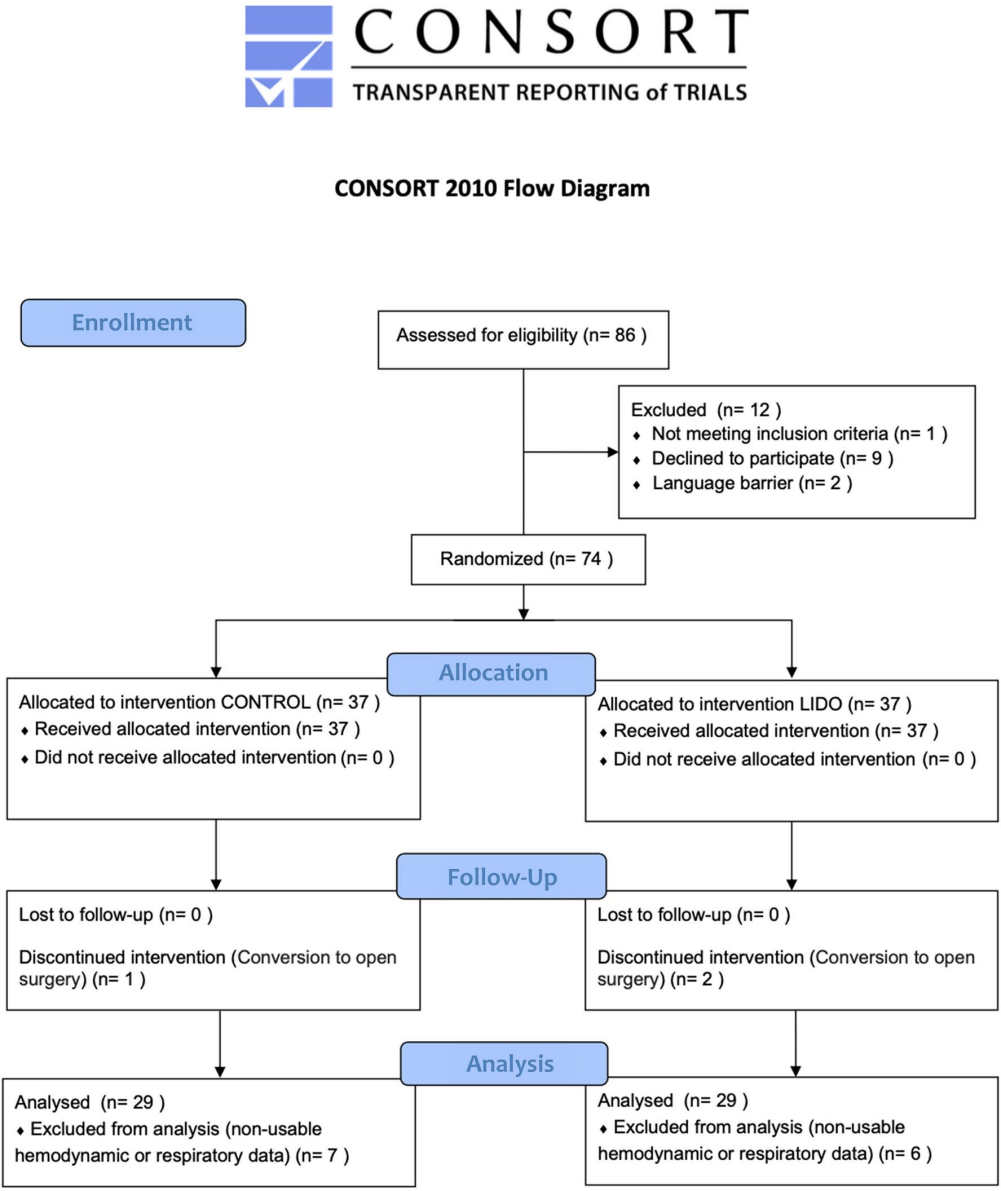
Flow chart of patients included in the study. Of the 74 patients enrolled, 58 were qualified for this study: 29 patients from the Lido group and 29 from the Control group. Hemodynamic parameters and lung compliance were available for 58 patients. However, P_peak_ and PEEP were registered in 38 patients (21 from the Lido group and 17 from the Control group).

From the recruited population, patients with complete respiratory module records were included in further analysis. Flow diagram of the study is presented in Fig. 1.

### Study intervention

Participants were randomly assigned to one of two groups: experimental (Lido) and control (Control).

Patients in the experimental arm received intravenous lidocaine bolus of 1.5 mg/kg over 5 min before induction of anesthesia followed by lidocaine infusion at 1.5 mg/kg/h intraoperatively. The infusion was discontinued before the patients’ transfer to the postanaesthesia care unit (PACU).

Patients in the control arm received no additional treatment.

Other than the studied intervention, both groups of participants were treated according to the same fixed perioperative care Protocol.

### Anesthesia protocol description

The peripheral intravenous catheter was inserted in the Emergency Room or in the Surgery Ward when obtaining blood samples. Topical local anesthetics were not used.

Due to the Principal diagnosis—acute appendicitis, all the cases were classified as emergencies (E).

Upon admission to the operating wing, IV midazolam at 0.05 mg/kg was administered for Premedication. The patient was then transferred to the operating theatre, where their vital signs were captured. In the lidocaine group, the loading dose of lidocaine was administered.

Induction of anesthesia was achieved with IV Propofol 4 mg/kg, fentanyl 3 μg/kg and rocuronium 0.6–1.2 mg/kg.

Standard monitoring of cardiopulmonary parameters and concentration of anesthetic gases and agents was utilized.

### Randomization

The eligible children were assigned to groups according to a computer-generated permuted block randomization list Prepared by a statistician with no clinical involvement in the trial.

The size of each block was six. Study allocation ratio was 1:1. Information about the participant’s allocation was concealed in a sealed envelope.

During the informed consent Process, the researchers, attending anesthesia teams and the children’s families were blinded to treatment allocation. After the consent had been obtained, the investigator opened the envelope. The study drug was Prepared by a nurse.

Throughout the study, all medication was administered to randomized patients according to their allocation.

All of the data analyzed in this report were collected during anesthesia.

### Study outcomes

Data on the use of medications and crucial moments such as intubation, skin incision, creation of pneumoperitoneum, end of the surgery, and extubation were collected in the operating theatre (OT) by the anaesthetist—one of the researchers.

Hemodynamic parameters were uploaded automatically to a database. Due to the lack of communication between the respiratory module and the server, those data were archived as photos.

### Outcome measures

Parameters were assessed at three stages of anesthesia. P_1_ corresponds to baseline readings, at the beginning of mechanical ventilation, P_2_—at the beginning of laparoscopic surgery after skin incision and pneumoperitoneum creation and readings made at the minimum of respiratory system compliance, and P_3_ corresponds to the end of surgery (end of pneumoperitoneum), before extubation.

- C—dynamic compliance of respiratory system, in ml/cmH_2_O;
- C/kg—pulmonary compliance per kilogram of body mass, in ml/kg/cmH_2_O;
- P_peak_—peak Pressure measured in the patient–ventilator system, in cmH_2_O;
- P_peak_-PEEP—the difference between peak Pressure and positive end-expiratory Pressure, in cmH_2_O;
- Pulse—pulse rate obtained by pulse oximetry, bpm;
- NBP_s_—systolic blood Pressure, in mmHg;
- NBP_d_—diastolic blood Pressure, in mmHg;
- NBP_m_—mean blood Pressure assessed as NBP_d_ + (NBP_s_-NBP_d_)/3, in mmHg;
- Correlations coefficients for dependencies: C vs. P_peak_, C vs. P_peak_-PEEP, C/kg vs. P_peak_, C/kg vs. P_peak_-PEEP, C vs. NBPs, C vs. NBP_d_, C vs. NBP_m_, C/kg vs. NBP_s_, C/kg vs. NBP_d_, C/kg vs. NBP_m_, C vs. Pulse, C/kg vs. Pulse.
- Curve equations for the above dependencies and R^2^ coefficients representing goodness of fit were determined, too.

### Statistical analysis

**Figure 2:**
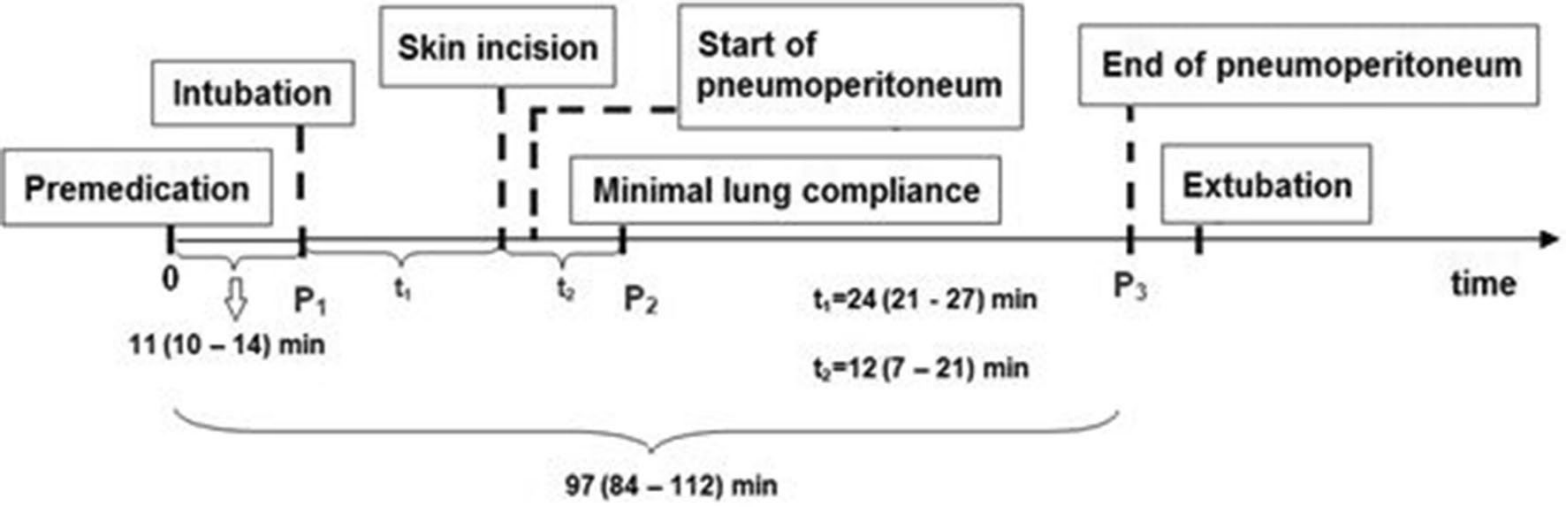
Study diagram showing key points of the operation: P_1_, P_2_, P_3_. The hemodynamic and respiratory parameters observed at these points were analyzed. Time data are expressed as the median and the range between the first and third quartile: Me (Q_1_–Q_3_).

The Shapiro–Wilk and Levene tests for normality and homogeneity of variance indicated that non-parametric tests should be performed in data analysis. Therefore, Mann–Whitney’s test was applied to evaluate the differences in respiratory (C, C/kg, P_peak_, P_peak_-PEEP) and hemodynamic parameters (NBP_s_, NBP_d_, NBP_m_, Pulse) between patient groups and to assess the difference between them at the successive stages of the operation: P_1_, P_2_, and P_3_. The Wilcoxon test was used to compare the P_1_ vs P_2_ and P_1_ vs P_3_ stages in the same patient group. Then, Friedman’s ANOVA test for repeated measurements was performed to assess the difference in these measures between the P_1_, P_2_, and P_3_ stages of the anaesthesia (Fig. 2).

Moreover, the Spearman correlation coefficient R_s_ and the R^2^ coefficient for goodness of fit of the equation curve to data distribution were determined, too. A P-value < 0.05 was considered to be statistically significant. All analyses were carried out with the Statistica software (StatSoft, Inc. (2011). STATISTICA (data analysis software system), version 10; www.statsoft.com.).

### Ethics approval and consent to participate

This study was approved by the Ethics Committee of the Medical University of Warsaw (KB/24/2019). The trial was registered at the US National Institutes of Health (ClinicalTrials.gov): NCT03886896. The date of registration: 15/03/2019. Written informed consent was Provided by parents/legal guardians.

## Results

Of the 71 participants who completed the original trial^1^, 58 were included in the current analysis, with 29 assigned to the lidocaine infusion group (Lido) and 29 assigned to the control group—without lidocaine infusion (Control).

Baseline demographic and clinical characteristics are Presented in Table 1.

**Table 1:** Patients’ characteristics.

Variable	Lido group N_1_ = 29	Control group N_2_ = 29
**Gender**		
Female/Male	9/20	10/19
**Age (years)**		
Median (Q_1_−Q_3_)	12 (9.3–12.6)	12 (8.7–14.1)
**Weight (kg)**		
Median (Q_1_−Q_3_)	38 (28–52)	40 (27–60)
**ASA Classification**		
IE/IIE	24/5	21/8

Q_,_ Q_3_—first and third quartile, Q_3_–Q_1_ = IQR (interquartile range), ASA—American Society of Anesthesiologists.

The Lido vs. Control group data did not differ statistically (P > 0.05)—the results of Chi^2^, and Mann–Whitney tests.

Lidocaine safety considerations were the major concern of the Primary trial. Frequencies of allergic reactions as well as neurotoxicity or cardiotoxicity episodes were specified secondary outcomes. None of the study participants developed signs of anaphylaxis, systemic toxicity, circulatory disturbances or neurological impairment.

**Figure 3:**
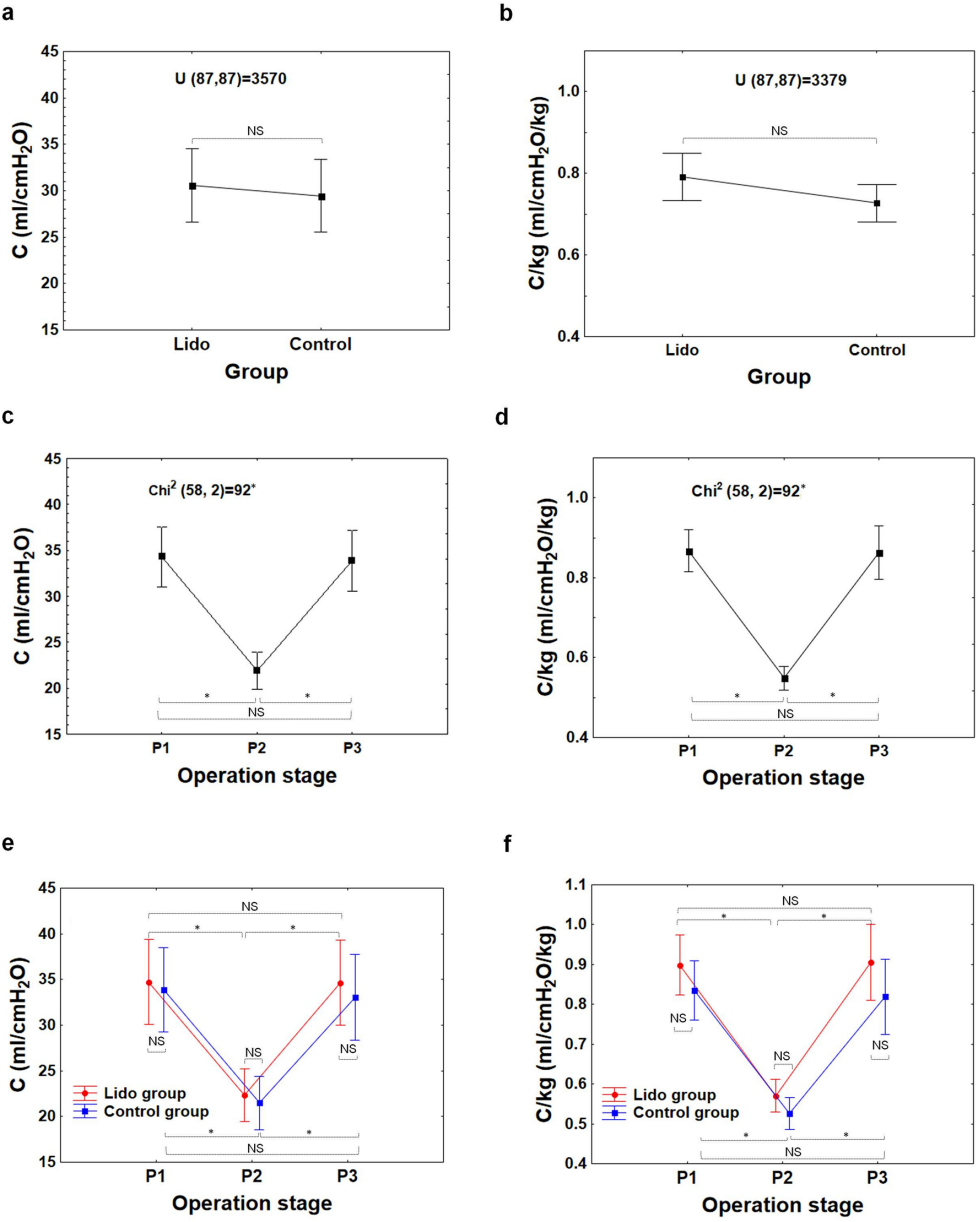
Compliance of respiratory system C, compliance of respiratory system per kg of body mass C/kg at the beginning (P_1_–baseline), during (P_2_) and at the end of surgery (P_3_) in the Lido and Control group. The differences between patients’ groups are insignificant (NS), (**a**, **b**). The differences between the P_1_, P_2_ and P_3_ stages are significant (**c**, **d**); *P < 0.001 (1 − β  = 0.99 for α = 0.001). The difference between Lido and Control group at the P_1_, P_2_ and P_3_ level is insignificant (NS), (**e**, **f**).

**Figure 4:**
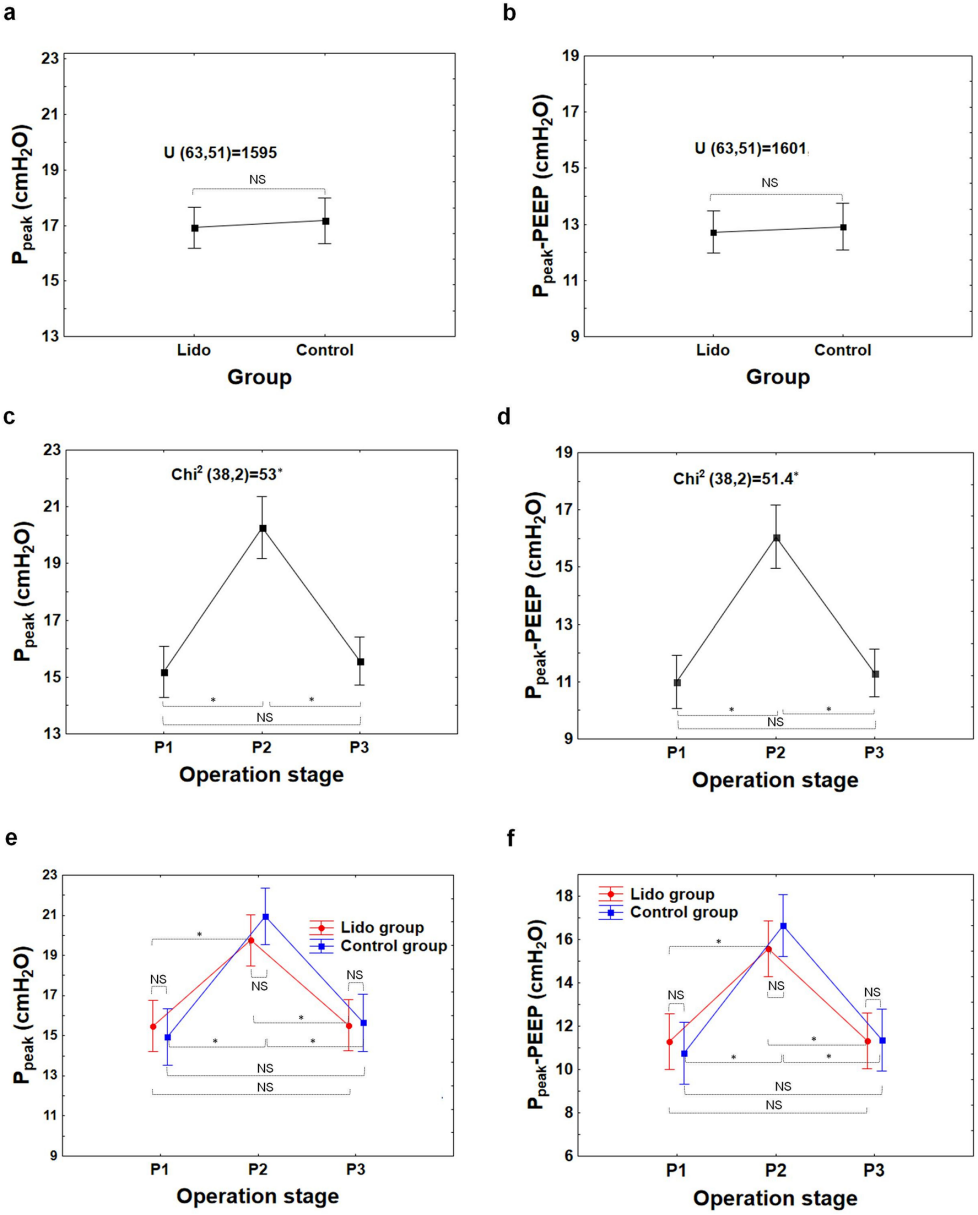
Peak pressure (P_peak_) and the peak pressure minus positive end-expiratory pressure (P_peak_-PEEP) in the patient-ventilator system at the beginning (P_1_−baseline), during (P_2_) and at the end of surgery (P_3_) in the Lido and Control group. The differences between patients’ groups are insignificant (NS), (**a**, **b**). The differences between the P_1_, P_2_ and P_3_ stages are significant (**c**, **d**); *P < 0.001 (1− β = 0.99 for α = 0.001). The difference between Lido and Control group at the P_1_, P_2_ and P_3_ level is insignificant (NS), (**e**, **f**).

**Figure 5:**
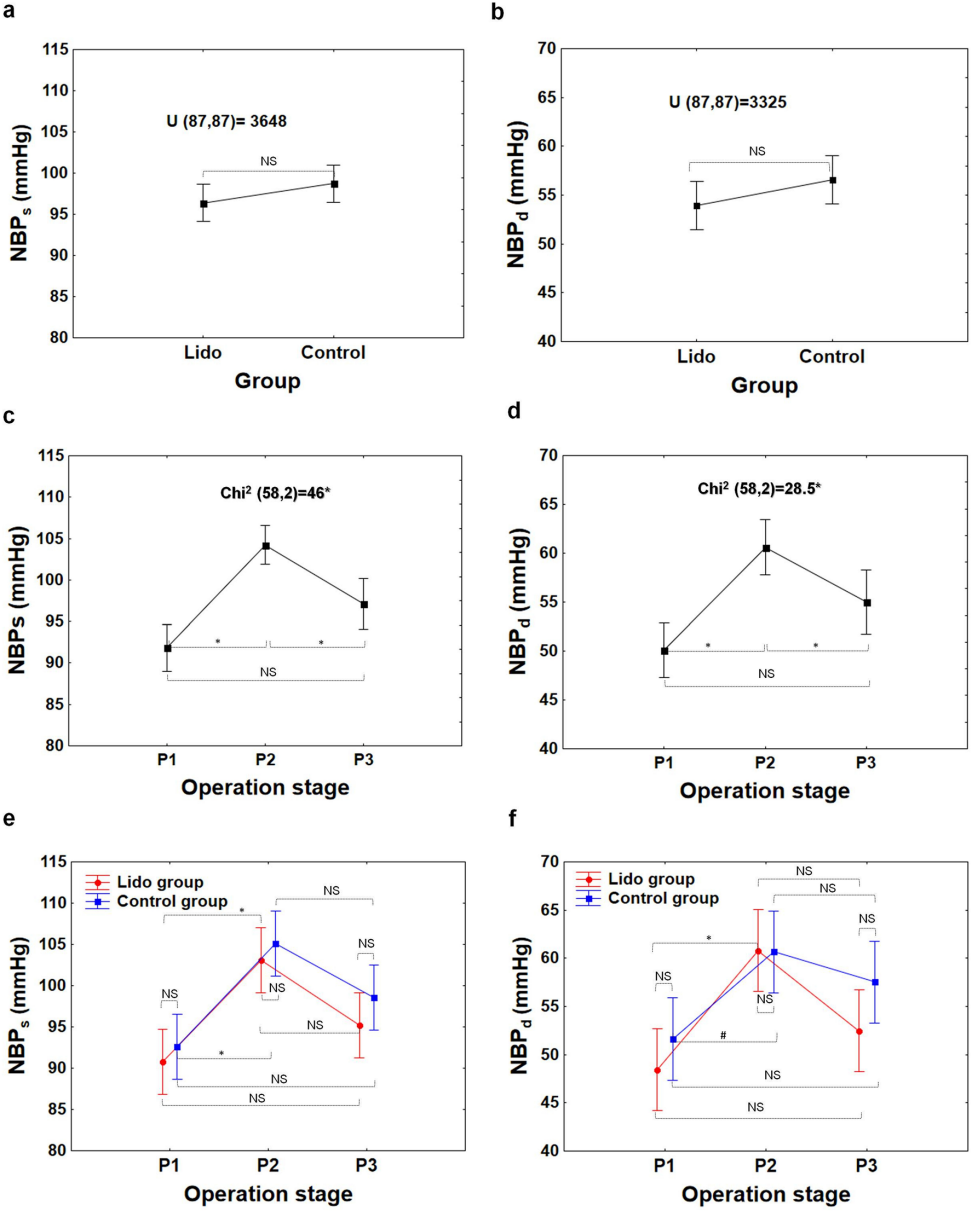
Systolic and diastolic blood pressure (NBP_s_, NBP_d_) at the beginning (P_1_−baseline), during (P_2_), and at the end of surgery (P_3_) in the Lido and Control group. The differences between patients’ groups are insignificant (NS), (**a**, **b**). The differences between the P_1_, P_2_ and P_3_ stages are significant (**c**, **d**); *P < 0.001 (1 − β = 0.99 and 1 − β = 0.98 for c and d respectively, at α = 0.001). The difference between Lido and Control group at the P_1_, P_2_ and P_3_ level is insignificant (NS), (**e**, **f**). ^#^P < 0.05.

**Figure 6:**
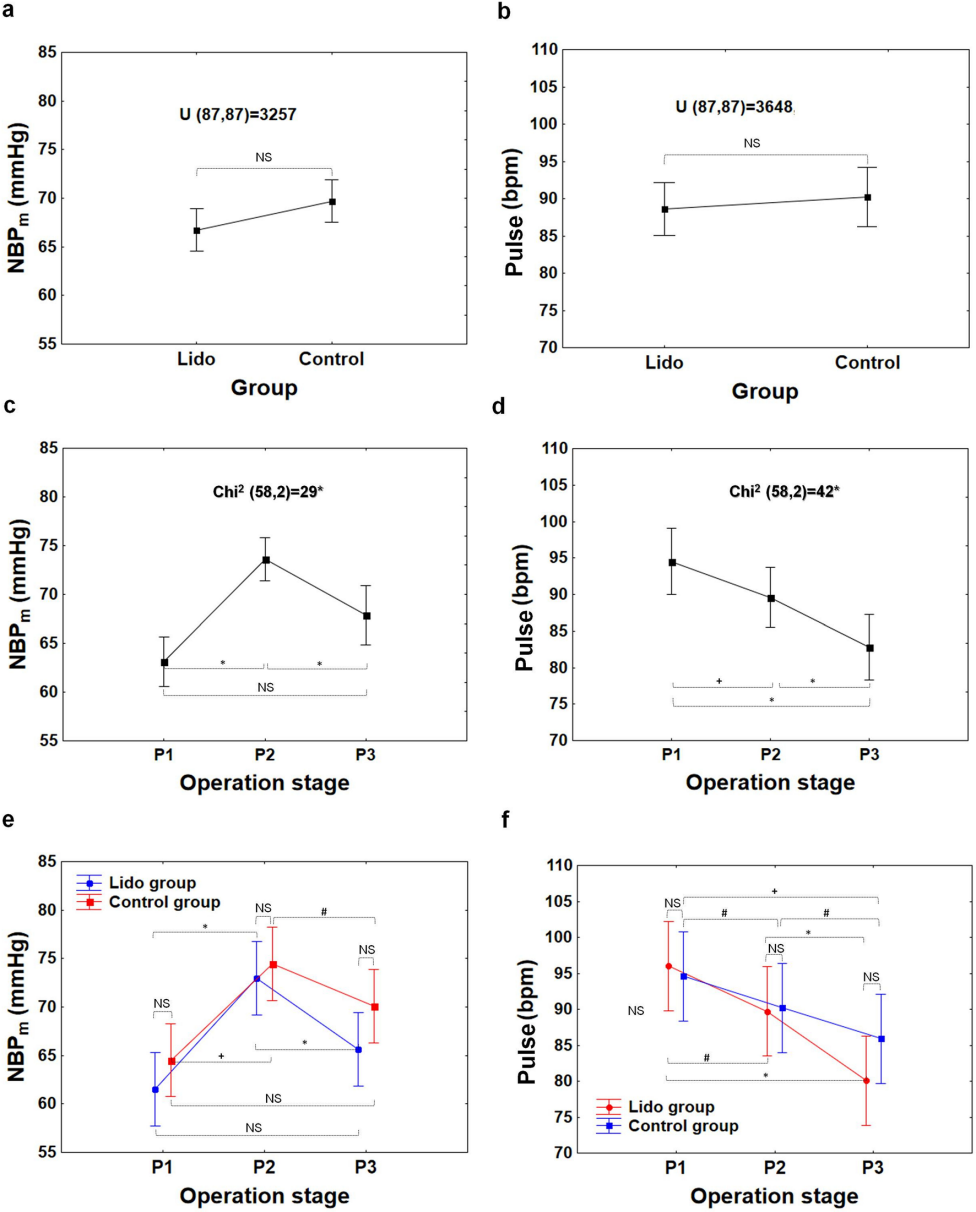
Mean blood pressure (NBP_m_) and pulse rate (Pulse) from SpO_2_, at the beginning (P_1_−baseline), during (P_2_), and at the end of surgery (P_3_) in the Lido and Control group. The differences between patients’ groups are insignificant (NS), (**a**, **b**). The differences between the P_1_, P_2_ and P_3_ stages are significant (**c**, **d**). *P < 0.001; 1 − β = 0.97 at α = 0.001 (**c**); 1 − β = 0.95 at α = 0.05 (**d**). The difference between Lido and Control group at the P_1_, P_2_ and P_3_ level is insignificant (NS), (**e**, **f**). ^**+**^P < 0.005, ^**#**^P < 0.05.

The statistical analyses of this study, concerning respiratory and hemodynamic parameters registered in patients during laparoscopic appendectomy, showed that there were generally no differences between the Lido and Control groups with respect to the following parameters: C, C/kg, P_peak_, P_peak_-PEEP, Pulse, NBP_s_, NBP_d_, and NBP_m_, at any point of examination (i.e.: P_1_, P_2_, or P_3_); see from Figs. 3a,b,e,f to 6a,b,e,f.

However, in both patient groups alike, the differences between measurements at P_1_, P_2_, P_3_ in all the variables assessed in the study were statistically significant (P < 0.001); Figs. 3c,d to 6c,d.

Among respiratory parameters, the C and C/kg values at the P_2_ level were significantly lower than those measured at P_1_ and P_3_ (Fig. 3c,d), whereas the values of P_peak_ and P_peak_-PEEP at that level were significantly higher than those observed at P_1_ and P_3_ (Fig. 4c,d); see also Tables S1–S2 (Supplementary information).

**Figure 7:**
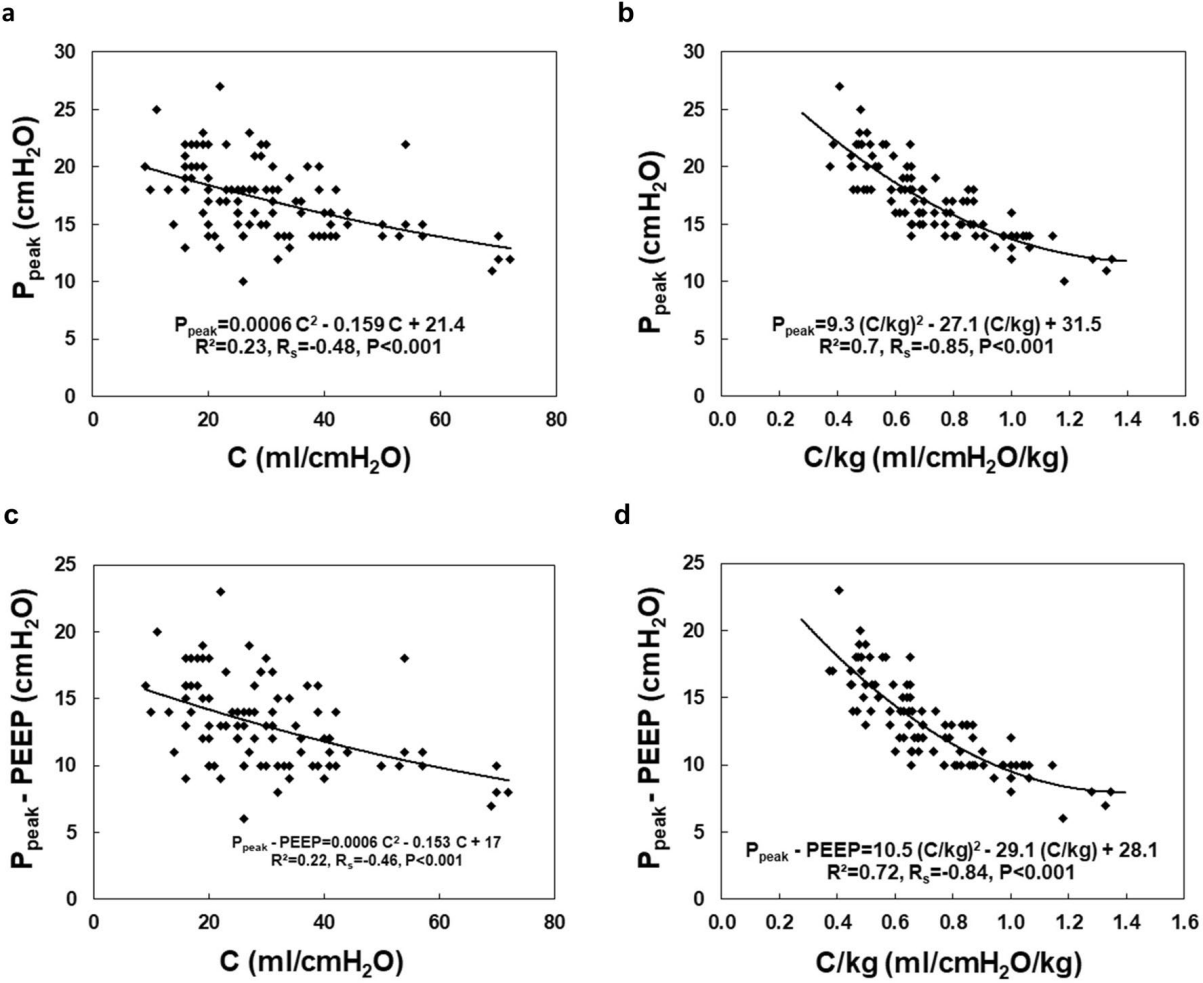
Correlation between peak pressure (P_peak_) and respiratory system compliance (C) (**a**), P_peak_ and the respiratory system compliance per kilogram of body mass (C/kg) (**b**), and between P_peak_-PEEP vs. C (**c**), and P_peak_-PEEP vs. C/kg (d). R_s_—Spearman correlation coefficient, R^2^—coefficient of determination. *P < 0.001.

Subsequently, moderate and significant correlations were noted between C and P_peak_ and C and P_peak_-PEEP; R_s_ = − 0.48 and R_s_ = − 0.46 respectively, P < 0.001 (Fig. 7a,c).

In terms of hemodynamic parameters received by all patients together, it was observed that NBP_s_, NBP_d_, and NBP_m_ at P_2_ were significantly higher compared to those assessed at the P_1_ and P_3_ (Figs. 5c,d, 6c); P < 0.001, 1− β = 0.9 ÷ 0.99 for α = 0.001). However, Pulse decreased with time in both patient groups, and the difference between P_2_ and P_1_ was significant (Fig. 6d,f); see Table S1 (Supplementary information). The Pulse at the end of surgery (P_3_) did not return to baseline (P_1_), and the difference was significant, both when the patient groups were considered together (P < 0.001, 1 − β = 0.84 at α = 0.01; Fig. 6d) and separately (Fig. 6f, Table S1). The test power values obtained for the “Operation stage” factor for all measured parameters were included in Table S2 (Supplementary information). Then, the exemplary curves of Pulse received during the operation in six patients from the Lido and Control group were presented in Figs. S1–S6 (Supplementary information).

The strongest significant correlations between the studied parameters were observed for the dependencies: C/kg vs P_peak_ (R_s_ = − 0.85, P < 0.001) and C/kg vs P_peak_-PEEP (R_s_ = − 0.84, P < 0.001), which are presented in Fig. 7b,d.

Moreover, moderate but significant correlations were found for C/kg vs NBPs (Rs = − 0.42, P < 0.001) and C vs Pulse, (Rs = − 0.48, P < 0.001), whereas weak but significant correlations were observed between: C/kg vs NBP_d_ (R_s_ = − 0.22, P < 0.01) and C/kg vs NBP_m_ (R_s_ = − 0.25, P < 0.001)—Supplementary information, Fig. S7a-d.

## Discussion

Lidocaine, a potent local anesthetic and class I B antiarrhythmic agent, is also recommended in perioperative medicine in the adult population as part of a multimodal approach to pain management^12,13^. At the same time, recommendations for children do not clearly support its use, and some have not even taken lidocaine into consideration^14,15^.

Despite the increasing popularity of systemic lidocaine administration in the pediatric population, due to the lack of clear evidence derived from randomized controlled trials, in Poland this intervention remains to be regarded as off-label use. Emerging publications report beneficial effects in pediatric patients, especially the opioid-sparing effect^1,3^, reduction in volatile anesthetic requirement^8,9^, and shorter hospital stays^3^.

The main objective of the Present analysis is to study systemic lidocaine infusions in terms of safety. The influence of lidocaine on changes in hemodynamic and respiratory parameters during abdominal laparoscopy was also investigated.

The paper also explores the physiological consequences accompanying laparoscopic Procedures in children.

The benefits of laparoscopic surgery include faster recovery, reduced postoperative pain, fewer wound-related complications and reduced morbidity^10^. Apart from the advantages associated with the surgical wound, there are also some potential complications that have to be taken into account. In this discussion, we address only the physiological consequences of pneumoperitoneum.

Without a doubt, laparoscopic Procedures affect circulatory and respiratory systems (Figs. 3, 4, 5, 6). The effect is dependent on patient characteristics (comorbidities, hydration), patient positioning, type of surgery, duration of Procedure, and intraperitoneal Pressure used.

Creation of pneumoperitoneum may Provoke a wide range of hemodynamic alterations, such as hypotension, hypertension, arrhythmias and even cardiac arrest. According to Gerges et al.^11^ intraabdominal Pressure (IAP), patient’s position, volume of carbon dioxide absorbed, patient’s intravascular volume, ventilatory technique, surgical conditions, and anesthetic agents used are the factors responsible for observed changes. In the adult population, IAP below 15 mm Hg increases cardiac output due to venous return reinforcement^11^. On the other hand, Perugini et al. state that cardiac output decreases while mean arterial Pressure remains unchanged or even increases up to 16%^16^.

Increase in IAP above the level of 15 mm Hg compresses vena cava inferior and collateral vessels resulting in decreased venous return, cardiac output and hypotension^10,11^.

Veress needle insertion and peritoneal stretch are some of the factors responsible for vagal stimulation leading to bradyarrhythmias. Tachyarrhythmias are attributed to hypercarbia and catecholamines^11^.

Our analysis is limited to children in ASA 1E/2E physical status Presenting for laparoscopic appendectomy in neutral or reverse Trendelenburg position with average intraperitoneal Pressure of 12 mmHg, not exceeding 15 mmHg.

In this study, the creation of pneumoperitoneum affected noninvasive blood Pressure (NBP_s_, NBP_d_, NBP_m_) (Figs. 5, 6). The change was strictly time-correlated with gas insufflation, and the effect was clinically and statistically significant (NBP_m_, median (Q_1_−Q_3_): P_1_: 61(55–72) mmHg, P_2_: 74(68–78) mmHg, P_3_: 68(61–73) mmHg; P < 0.001). The most plausible physiological explanation of this phenomenon is that positive IAP shifts the blood from minor visceral capillaries to larger vessels, increasing cardiac Preload. According to the Frank–Starling law, an increase in the end diastolic volume Produces an increase in stroke volume.

None of the patients experienced the reverse effect, which is a potential complication of laparoscopic Procedures in certain groups of patients (i.e. with heart failure, hypovolemic), especially when IAP exceeds 15 mm Hg.

This study did not reveal any statistically significant differences between NBP in the experimental and the control group (Figs. 5, 6).

While NBP is strictly correlated with the presence of pneumoperitoneum, changes in the Pulse rate seemed to be more independent. The Pulse decreased as the surgery progressed (Fig. 7).

In the course of CO_2_ insufflation, the decrease in Pulse might be explained by the baroreceptor reflex. Then, during the stable phase of surgery, a slight increase in Pulse up to a plateau level was observed in some patients, reflecting adaptation to altered hemodynamic conditions (Supplementary information, Fig. S1–S6). The end of peritoneal oedema cessation was followed by a significant decrease in Pulse, too, which might be explained by the Bainbridge effect. The two physiological effects are in opposition to each other, and the balance between them ensures Pulse stability. The switch of Predominance from one to the other in course of laparoscopic surgery is difficult to elucidate.

There were no incidents of hemodynamic safety threshold braking. The preliminary conclusion is that in the child population at ASA class 1E, 2E undergoing laparoscopic appendectomy with IAP limited to 15 mmHg, the observed cardiovascular alterations, although statistically significant, are predictable and harmless. No significant impact of lidocaine infusion on hemodynamic reaction to pneumoperitoneum creation was observed.

Finally, since no significant differences between groups were found with respect to Pulse or NBP (Figs. 5, 6), the systemic lidocaine infusion in the studied regimen appears not to have cardiodepressive Properties.

During the laparoscopic procedure, due to pneumoperitoneum creation, the predictable respiratory changes were also observed. Pulmonary compliance, both absolute (C, in ml/cmH_2_O) and relative (C/kg, in ml/cm H_2_O/kg), decreased significantly (Fig. 3). In consequence, increased pressures of ventilation P_peak_ and P_peak_-PEEP were needed (Figs. 4, 7). This effect is of clinical and statistical importance (Fig. 7, Table S1—Supplementary information). The main cause of the above phenomenon is an increase in IAP, which causes a diaphragm elevation resulting in the restriction of lung distention. Neuro-endocrine response due to increased IAP triggers an activation of the renin–angiotensin–aldosterone system (RAAS), increased antidiuretic hormone secretion and vagal reflexes^19^. The RAAS affects both the circulatory system and lungs thus regulates immune-inflammatory response, and other mechanisms contributing to lung injury or even different pulmonary diseases^20^. Due to this reaction, the resistance of respiratory system may increase correspondingly. Beaussier et al., in their review article^17^ mentioned several trials investigating the influence of intravenously administered lidocaine on the respiratory system. According to them, lidocaine as an agent with anti-inflammatory properties, exhibiting relaxant effect on tracheal smooth muscle cells and inhibiting bronchial hypersensitivity, was examined for compliance-preserving abilities. Our analysis does not support this hypothesis in our specific circumstances. Intravenous lidocaine infusion did not affect respiratory parameters like: C, C/kg, and thus offered no protection against increased need for P_peak_, P_peak_-PEEP utilization. The time correlation between pneumoperitoneum creation and decrease in pulmonary compliance is clear (see Figs. 3c, d and 4c, d). Unfortunately, due to the lack of continuous IAP recordings, this analysis did not lend itself to establishing the relationship between an increase in intraperitoneal pressure and a decrease in compliance. Thus, the question whether the relationship is universal or patient-specific remains open.

According to the current analysis, continuous intravenous lidocaine infusion appears to be a safe modality, which is consistent with other publications^2–9,17^. In addition to supporting the safety of systemic lidocaine, our paper presents an in-depth analysis of cardiopulmonary reactions in children.

### Study limitations

The Primary study^1^ was designed to assess the influence of lidocaine infusion on: opioid requirement during the first 24 h after surgery, intraoperative fentanyl consumption, intraoperative sevoflurane consumption, time to the first rescue analgesic request, incidence of postoperative nausea and vomiting during the first 24 h after surgery, frequency of side effects of lidocaine. The study was not designed primarily with a view to evaluating the influence of lidocaine infusion on respiratory and circulatory parameters.

None of the enrolled patients was in ASA physical status class greater than 2E, hence even if respiratory disease affected a fraction of study participants, it was mild and without substantive functional limitations for them. Regardless of this fact the omission of respiratory disease occurrence in study eligibility criteria is concerned a study limitation.

Another limitation is the lack of data describing pneumoperitoneum parameters. The rates of carbon dioxide insufflation and intraperitoneal pressure were measured constantly, but not recorded hence they were not available to analyze correlations with respiratory and circulatory parameters.

The only known data are the time of pneumoperitoneum creation, which was written down, and the fact that the average Pressure was approximately 12 mmHg and the threshold of 15 mmHg was not exceeded, in accordance with a standard surgical Protocol.

In 2015, Amato et al.^18^ found that driving Pressure was strongly associated with survival in patients with the acute respiratory distress syndrome (ARDS). Since then, the parameter has been investigated both as a Predictor of mortality and as a guidepost in lung Protective strategies. Driving Pressure (∆P) is calculated as plateau Pressure (P_plat_) minus positive end-expiratory Pressure (PEEP), or can be expressed as the ratio of tidal volume (Vt) to respiratory system static compliance (C_stat_). Unfortunately, due to the characteristics of the anesthetic device plateau pressure and static compliance were not available, that is why ∆P could not be calculated. P_peak_ reflects lung compliance and airway resistance, PEEP was constant throughout the surgery, therefore P_peak_−PEEP seems to be an adequate tool for assessing the overall respiratory reaction to pneumoperitoneum, which was one of the objectives of the study.

## Conclusions

Although an increase of IAP up to 12 (max. 15) mmHg during laparoscopic appendectomy causes significant changes in hemodynamic and respiratory parameters, patients in ASA classes 1E, 2E appeared not to be at risk of experiencing catastrophic reactions. Systemic lidocaine infusions do not alleviate circulatory and respiratory alterations during pneumoperitoneum creation and maintenance.

## Supplementary Information

Supplementary Information.

## Data Availability

The datasets used and/or analyzed during the current study are available from the corresponding author on reasonable request.
