# Supplementary material for: Impact of lidocaine on hemodynamic and respiratory parameters during laparoscopic appendectomy in children
Source: Sci Rep. 2022 Aug 18;12:14038. doi: 10.1038/s41598-022-18243-3 (PMC9388633; doi:10.1038/s41598-022-18243-3)
Supplement: Supplementary file 1 — Supplementary Information. [file 41598_2022_18243_MOESM1_ESM.docx]

Supplementary information


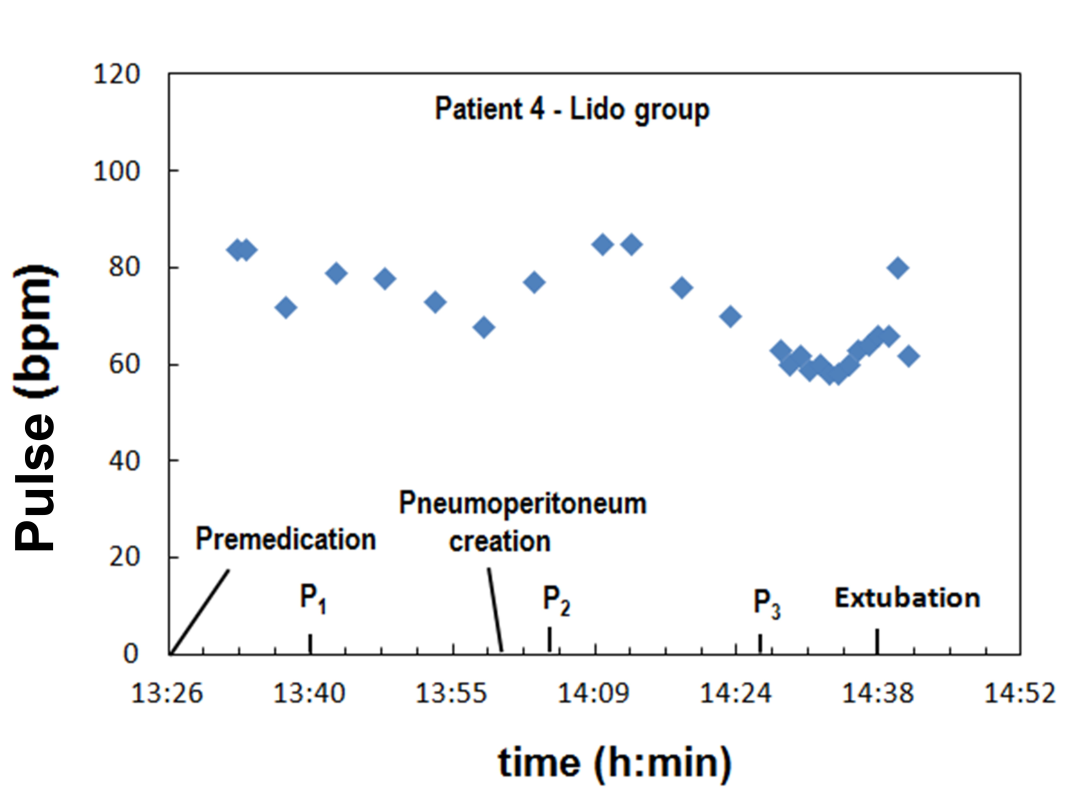


**Figure S1.** Pulse rate (Pulse) during the operation in patient 4 (Lido group). P_1_ – intubation, P_2_ – minimum of respiratory system compliance, P_3_ – the end of operation.


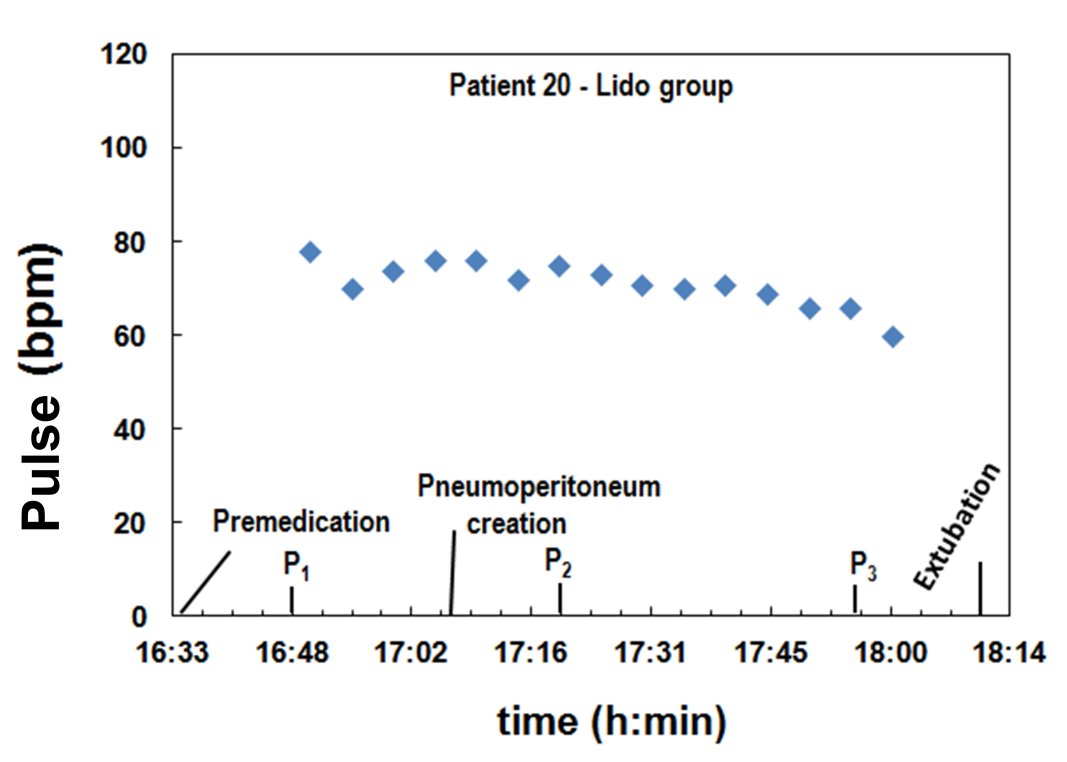


**Figure S2.** Pulse rate (Pulse) during the operation in patient 20 (Lido group). P_1_ – intubation, P_2_ – minimum of respiratory system compliance, P_3_ – the end of operation.


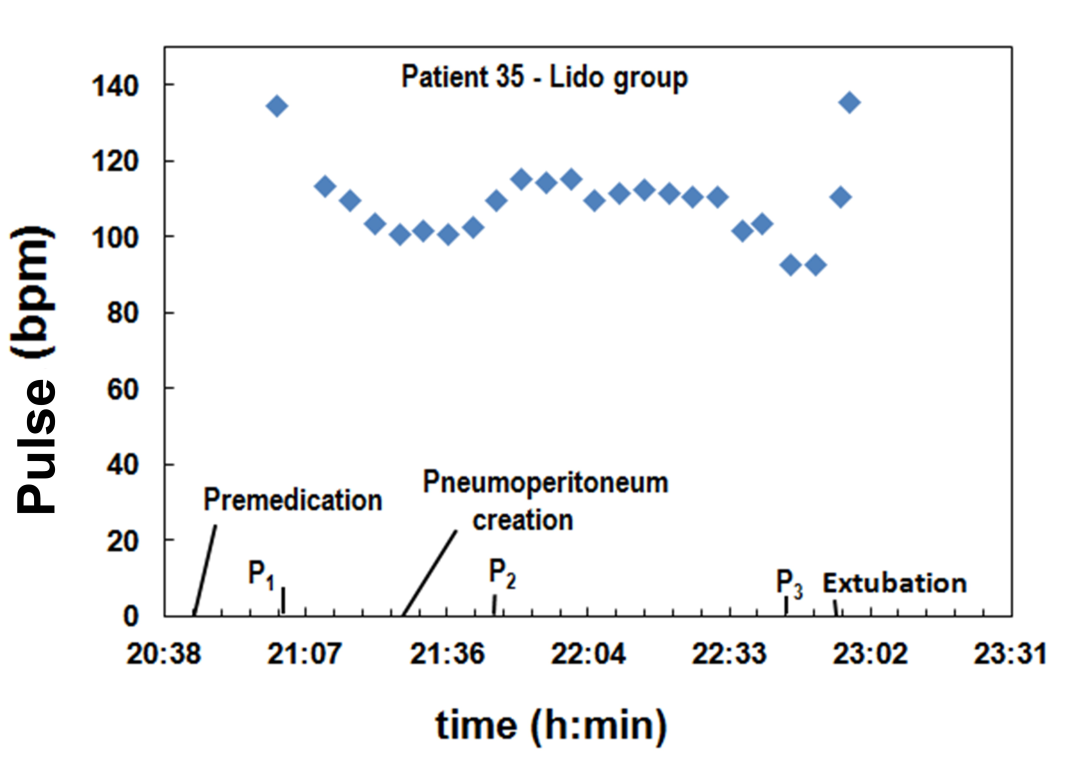


**Figure S3.** Pulse rate (Pulse) during the operation in patient 35 (Lido group). P_1_ – intubation, P_2_ – minimum of respiratory system compliance, P_3_ – the end of operation.


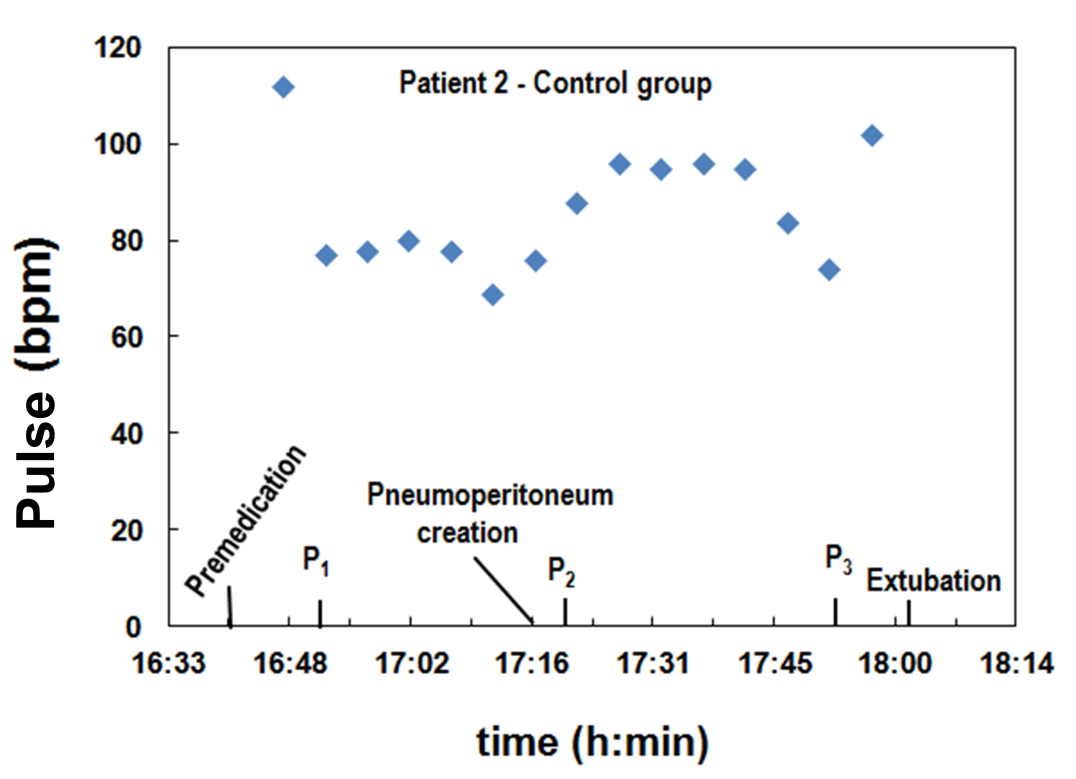


**Figure S4.** Pulse rate (Pulse) during the operation in patient 2 (Control group). P_1_ – intubation, P_2_ – minimum of respiratory system compliance, P_3_ – the end of operation.


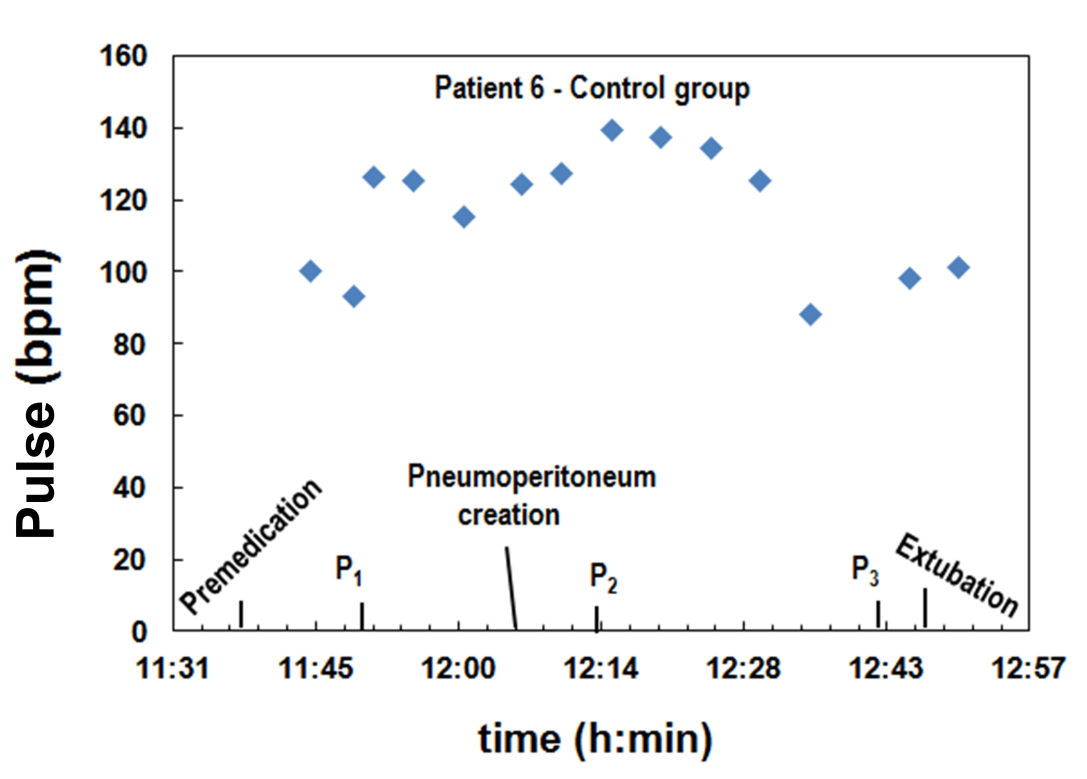


**Figure S5.** Pulse rate (Pulse) during the operation in patient 6 (Control group). P_1_ – intubation, P_2_ – minimum of respiratory system compliance, P_3_ – the end of operation.


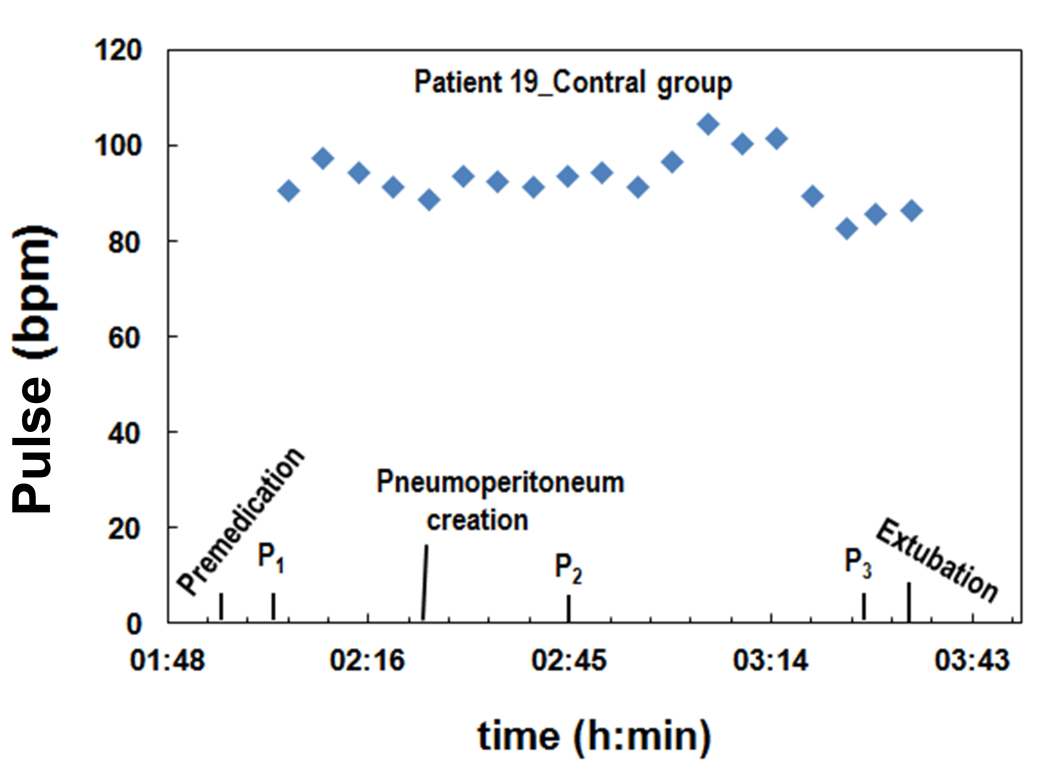


**Figure S6.** Pulse rate (Pulse) during the operation in patient 19 (Control group). P_1_ – intubation, P_2_ – minimum of respiratory system compliance, P_3_ – the end of operation.


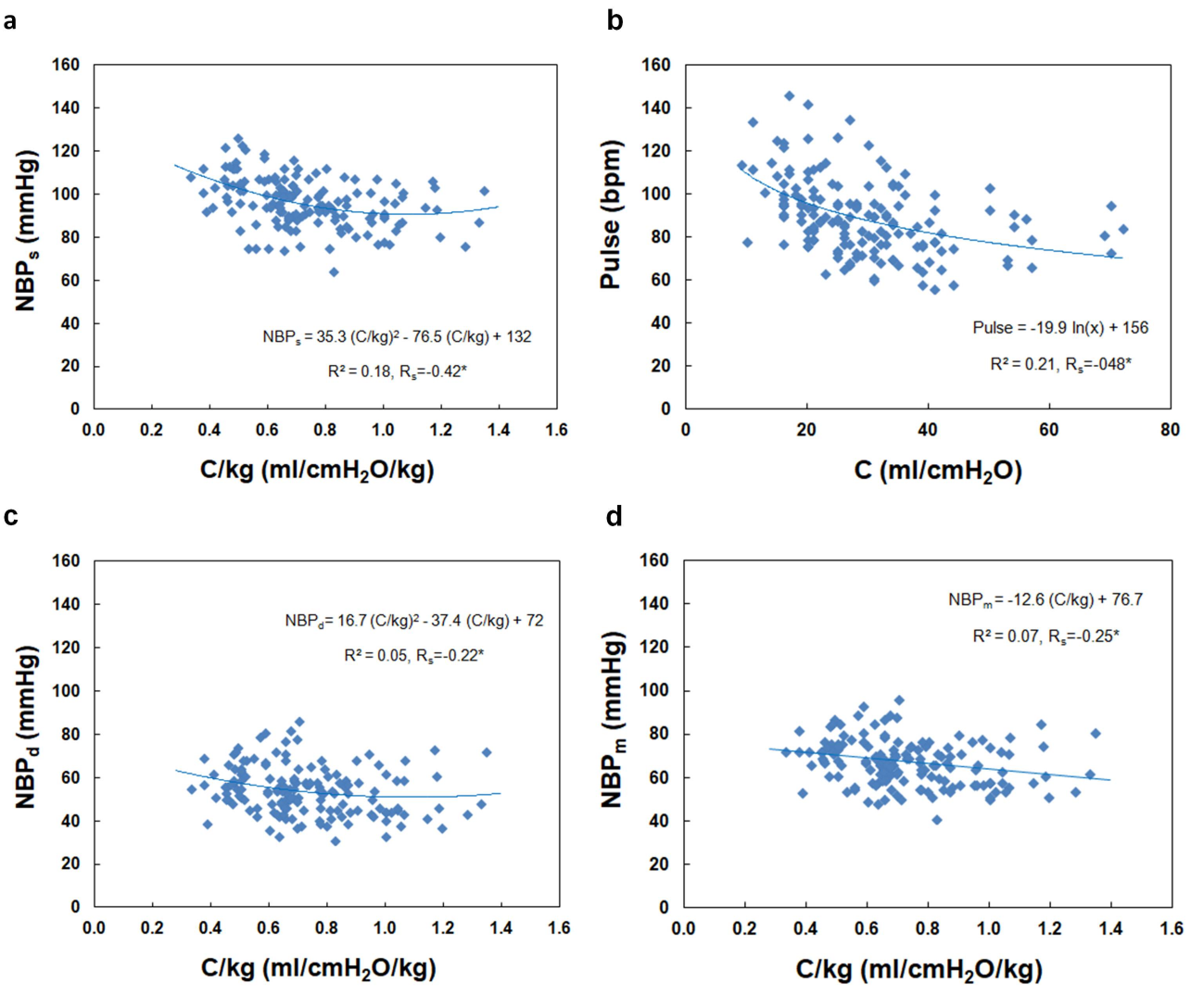


**Figure S7.** Correlation of systolic, diastolic, mean blood pressure (NBP_s_, NBP_d,_ NBP_m_) and Pulse (SpO_2_) with respiratory system compliance per kilogram of patient body mass (C/kg) (a-d). R^2^ – coefficient of determination R_s_ – Spearman correlation coefficient, *P<0.001.

**Table S1.** Hemodynamic and respiratory parameters obtained at the P_1_, P_2_ and P_2_ points of laparoscopic appendectomy in Lido and Control group of children.

| **Parameters** | **Lido group** | | | | **Control group** | | | |
| --- | --- | --- | --- | --- | --- | --- | --- | --- |
|  | **P_1_** | **P_2_** | **P_3_** | **overall** | **P_1_** | **P_2_** | **P_3_** | **overall** |
| NBP_s_  (mmHg) | 89  (83-99) | 102  (97-107)* | 95  (90-100) | 96  (89-104) | 92  (84-99) | 104  (101-112)* | 97  (92-101) | 99  (92-106) |
| NBP_d_  (mmHg) | 46  (43-56) | 57  (53-68)* | 53  (46-58) | 53  (46-60) | 49  (41-59) | 59  (55-66) | 57  (49-61) | 57  (48-64) |
| NBP_m_  (mmHg) | 58  (55-69) | 71  (67-77)* | 66  (60-70) | 67  (59-72) | 63  (55-73) | 74  (69-78)* | 69  (64-74) | 70  (62-76) |
| Pulse  (bpm) | 95  (85-105) | 84  (78-102)^#^ | 80  (67-90)^*, *^ | 87  (77-100) | 91  (82-105) | 91  (77-98)^#^ | 83  (74-94)^#,+^ | 90  (76-99) |
| C (ml/cmH_2_O) | 32  (27-38) | 21  (17-25)* | 32  (27-40) | 28  (22-36) | 33  (25-39) | 20  (16-27)* | 31  (21-39) | 28  (20-35) |
| C/kg (ml/cmH_2_O/kg) | 0.93  (0.69-1.02) | 0.59  (0.46-0.65)* | 0.84  (0.73-0.98) | 0.73  (0.62-0.96) | 0.83  (0.68-0.95) | 0.5  (0.48-0.59)* | 0.81  (0.69-0.84) | 0.73  (0.58-0.84) |
| P_peak_ (cmH_2_O) | 14  (13-17) | 20  (18-21)* | 15  (14-17) | 17  (14-20) | 15  (13-16) | 20  (18-22)* | 15  (14-17) | 16  (14-19) |
| P_peak_-PEEP (cmH_2_O) | 10  (9-12) | 16  (14-17)* | 11  (10-13) | 12  (10-16) | 10  (9-12) | 16  (14-17)* | 11  (10-13) | 12  (10-14) |
| The data are Median (Q_1_-Q_3_), where Q_1_=first quartile, Q_3_=third quartile, Q_3_-Q_1_=IQR, IQR – interquartile range.  *P<0.001 for P_2_ vs. P_1_ and P_3_ (excl. Pulse), P_1_ vs. P_3_ – not significant (excl. Pulse); Pulse (both groups together): ^+^P<0.005 for P_1_ vs P_2_ and *P<0.001 for P_2_ vs P_3_ and P_1_ vs P_3_; Pulse (Lido): #P<0.05 for P_1_ vs P_2_ and *P<0.001 for P_2_ vs. P_3_ and P_1_ vs P_3_; Pulse (Control): ^#^P<0.05 for P_1_ vs P_2_ and P_2_ vs P_3_, ^+^P<0.005 for P_1_ vs P_3_. The comparison results received using Friedman’s ANOVA, Mann-Whitney and Wilcoxon tests. | | | | | | | | |

.

**Table S2.** The test power (1-β) for the Operation stage factor *.

|  | **α=0.05** | **α=0.01** | **α=0.001** |
| --- | --- | --- | --- |
| **C** | 0.999 | 0.999 | 0.999 |
| **C/kg** | 0.999 | 0.999 | 0.999 |
| **P_peak_** | 0.999 | 0.999 | 0.999 |
| **P_peak_-PEEP** | 0.999 | 0.999 | 0.999 |
| **NBP_s_** | 0.999 | 0.999 | 0.994 |
| **NBP_d_** | 0.995 | 0.976 | 0.895 |
| **NBP_m_** | 0.999 | 0.995 | 0.969 |
| **Pulse** | 0.946 | 0.837 | 0.603 |
| *P<0.001 | | | |
